# Prosperine and Dr. Malchow’s Book

**Published:** 1909-03

**Authors:** E. C. Margrander

**Affiliations:** 623 Portland Avenue, Rochester, N. Y.


### Correspondence.

### Prosperine and Dr. Malchow's Book.

ROCHESTER, N. Y., February 4, 1909.

*F. E. Daniel, Editor Texas Medical Journal.*

DEAR DOCTOR: Although I reside several thousand miles away from the Lone Star State, the distance does not, however, prevent my expressing to you through a letter my admiration for your dauntless defense of independent medical journalism as against that variety which uses the organization mask to hide itself and its questionable methods and tactics.

I find the "Red Back" in the medical libraries in New York City, where I am during the greatest part of the year, engaged in research, therapeutic investigation and allied work. I need not assure you of my entire sympathy with your uniform policy of open, frank and honest speech and action. It is refreshing in these days, where principle is constantly sacrificed to policy, to find a few sturdy physicians battling for the right, regardless of bureaucratic organization and power seemingly entrenched so firmly that individual protests meet only derisive ridicule.

I have been watching month by month for the promised review of Dr. Malchow's book, "Sexual Life," by "Mrs. Proserpine." As this review did not appear in the December issue, I presume that it is contained in the January number, which I have not been able to find in this city (Rochester), where I have passed the holiday season. Will you, therefore, send me two copies of the issue in which Mrs. Proserpine's review has appeared. Regarding Dr. Malchow, it seems a disgrace to the medical profession of the Middle West that not one society came to his aid when he was attacked by these well known individuals, who have in the past, and do yet, move heaven and earth to prevent, now among physicians, the dissemination of most necessary knowledge regarding the sex function and sex relation. In one session of the New York branch of the American Society for Social and Moral Prophylaxis, of which the eminent Dr. Prince Morrow is President, this question was brought up by a prominent attorney, who stated that the time would come, sooner or later, when the medical profession would be compelled to cross swords with the repressing influences whose activities have produced a little good but incalculably more harm.

Hoping that you will continue in your good work, I am,

Fraternally yours,

E. C. Margrander,

623 Portland Avenue, Rochester, N. Y.

